# P355: Quality of the management tray metrological technical clinical biochemistry public laboratory

**DOI:** 10.1186/2047-2994-2-S1-P355

**Published:** 2013-06-20

**Authors:** M Adeoti, C Lodjou

**Affiliations:** 1 Centra Laboratory, CHU of Yopougon, Abidjan, Côte d'Ivoire; 2 Health Ministry , Cotonou, Benin

## Introduction

Because of its role as provider of services for clinicians and their identification with the central tool for the production of the hospital, the technical platform that is the hospital laboratory and its equipment requires the development of quality assurance.

## Objectives

Evaluate the quality of the metrological management of the technical elements with a view to continuous improvement of the services provided.

## Methods

It is a cross-sectional study and prospective descriptive type conducted over a period of three months in clinical biochemistry laboratories of three university hospitals in Abidjan. Performed using a plug-inventory survey of twelve open-ended questions, it focused on the environmental conditions and management of the technical platform. The performance of the laboratory was been assessed against the threshold target of 80% expected the quality standard used.

## Results

Averages of 67% for environmental conditions and 42.77% for equipment and instruments were observed. Only 48.15% of the equipment and measuring instruments of the three laboratories are functional, with a better score for the CHU of Cocody. The laboratory of the University Hospital of Yopougon appears better informed on the equipment and measuring instruments (78%). Regular maintenance and the maintenance of the technical concerns only 13.33% of the equipment and measuring instruments which are less than 15% subject to a procedure known fault management operation related to.

## Conclusion

These results show notable deficiencies in the practice of primary metrological activities to guarantee the reliability of the final product laboratories, and therefore emphasize the need for development of a strategy to regulate the technical tools of analysis in the context of a quality assurance process.

## Disclosure of interest

None declared.

